# Institutional delivery services utilization and its determinant factors among women who gave birth in the past 24 months in Southwest Ethiopia

**DOI:** 10.1186/s12913-020-05121-9

**Published:** 2020-03-30

**Authors:** Metsehet Yoseph, Solomon Mekonnen Abebe, Fantahun Ayenew Mekonnen, Mekonnen Sisay, Kedir Abdela Gonete

**Affiliations:** 1 Department of Reproductive Health, Institute of Public Health, College of Medicine and Health Sciences, University of Gondar, Gondar, Ethiopia; 2 Department of Human Nutrition, Institute of Public Health, College of Medicine and Health Sciences, University of Gondar, Gondar, Ethiopia

**Keywords:** Institutional delivery, Postnatal mothers, Factors, Ethiopia

## Abstract

**Background:**

Institutional delivery is a delivery that takes place at any medical facility staffed by skilled delivery assistance. It is estimated that using institutional delivery could reduce 16 to 33% of maternal deaths. Despite the importance of delivering at health institutions, in Ethiopia, mothers prefer to give birth at home. Therefore, the aim of this study was to compare institutional delivery service utilization and associated factors among rural and urban mothers in Mana district, Jimma Zone, Southwest Ethiopia, 2017.

**Methods:**

A community based comparative cross sectional study was conducted in Mana district, Southwest Ethiopia from March to June, 2017. A stratified probability sampling technique was used to select a total of 1426 (713 urban, 713 rural) study participants. Data were collected from mothers who gave birth in the past 24 months by using a structured and pretested questionnaire.

**Results:**

The overall prevalence of institutional delivery service utilization was 86.4%. Higher number of antenatal care visits, having good knowledge on the danger signs of labor, increased wealth index, primary and above educational level of the husband, mothers age below 40-year, and less than 30-min travel time to the nearby health institutions had significantly increased the rate of institutional delivery service utilization.

**Conclusion:**

Institutional delivery service utilization is high in the study area. Maternal awareness of danger signs of labor, frequent ANC visit and better education of husband, and household wealth predicted the utilization of the service. Therefore, due attention need to be given to enhancing education, expanding health institutions and creating awareness on advantage of antenatal care follow up and danger signs to make all pregnancies delivered at health institutions.

## Background

Institutional delivery is a delivery that takes place at any medical facility staffed by skilled delivery assistant (www.cdedse.org/ws2011/papers/Ambris). Institutional delivery service utilization is one of the key and proven intervention to improve maternal health and wellbeing and to reduce maternal mortality through providing safe delivery and reducing complications that are related to and occurred during birth [1].

It is well known that complications which occurred during labor, delivery and the immediate postpartum period are responsible for the majority of maternal deaths [2]. To reduce the level of maternal mortality, the World Health Organization (WHO) envisions a world where “every pregnant woman and newborn receive quality care throughout pregnancy, childbirth and postnatal period” [3]. The safe motherhood initiative also highly emphasizes institutional delivery as one element of emergency obstetric care where complicated cases can be safely handled [4]. It is estimated that using institutional delivery could reduce 16 to 33% of maternal deaths [5, 6].

However, due to low utilization of institutional delivery, maternal mortality remains as a major challenge in Ethiopia [7]. Therefore, promoting health institution delivery with a referral capacity is the most efficient strategy to reduce maternal deaths for lower-income countries including Ethiopia [8].

About 81% of urban and 61% of rural mothers had institutional delivery worldwide. Regarding to Sub Saharan countries, 75% of urban and 40% of rural mothers have given birth at health institutions [9]. In Ethiopia, utilization of institutional delivery services among pregnant women is found to be the lowest compared to the global report as only, 26% of deliveries had taken place at the health institutions [7, 10]. The majority of deliveries (80%) were taken place in the urban health institutions and only 21% were in the rural once, and 19% in Oromia region (where this study was conducted) [7].

Different researchers around the world and in Ethiopia have identified socioeconomic, socio demographic factors and lack of infrastructure facilities [11, 12], health facility factors, obstetric factors, poor maternal decision power on birth places, poor knowledge about the benefits of institutional delivery, cultural and traditional factors as barriers of institutional delivery service utilization [10, 13]. Whereas, residence, ANC, health education, decision on place of delivery with partners, maternal education level and knowledge of mothers on pregnancy and delivery services, family size, availability of transport, planned pregnancy, and participation of women monthly health conference are the factors that enhance institutional delivery services utilization [14–16]..

Institutional delivery remains low both regionally and nationally. This could probably be due to the fact that lack of evidences regarding the possible bottlenecks in the implementation of efforts employed to increase institutional delivery so far. Therefore, this study aimed at assessing institutional delivery service utilization and its associated factors among urban and rural women who gave birth in the past 24 months in Manna district, Southwest Ethiopia.

## Methods

### Study design and setting

A community based comparative cross sectional study was conducted on women in Mana district from March to June, 2017. Mana district, found in Oromia Regional State Southwest Ethiopia, is located 352 km far from Addis Ababa, capital city of Ethiopia. The district has a total of 191,608 inhabitants of whom 42,402 were women of reproductive age. According to the information obtained from Mana district health office, there are 26 kebeles (2 urban and 24 rural) and 42 health facilities (7 health centers and 35 health posts). About half (50.7%) of pregnant mothers received antenatal care from a skilled provider, 30% took iron tablets, and 5.1% of them took intestinal parasite drugs. A small proportion (0.2%) of women have experienced obstetrical fistula, 18% of women were thin and 27.3% had any form of anaemia. Children with any form of anaemia were 65.5%, and there were 37% neonatal, 60% infant, 20% child, and 79% under-5 mortality rates in the study region [7].

### Study population, sample size determination, and sampling procedure

All women who gave birth in the past 24 months in the randomly selected urban and rural kebeles were included in this study. Women who were seriously ill or unable to communicate were excluded.

The sample size for the prevalence objective was calculated using a two population proportion formula, considering the following assumptions; 10.2 and 58.5% prevalence (P) of institutional delivery among rural and urban women, respectively [9], 95% confidence interval, 80% power and 5% margin of error. To determine the sample size for the second objective factors like ANC visit, obstetric complication and distance to the nearest health institution were considered for the rural population [13], whereas, factors like place of residence, informed about place of delivery during pregnancy and knowledge of mothers about institutional delivery were used for the urban population. A sample size of 30 was computed for each population for the first objective, while 432, the largest sample, was obtained for each population for the second objective when distance from the nearest health institution was taken as predictor variable [13] . After multiplying this sample size with a design effect of 1.5 and adding 10% non-response rate, the total sample size was 1426 (713 for urban and 713 for rural).

A multi-stage sampling technique was employed to select the study participants. The district was classified into two strata, urban and rural, and out of 26 kebeles, two urban (namely Yebu and Belida), and 5 rural kebeles (namely Meti, Buture, Hunda toli, Dawa and Lemi Lelisa) were randomly selected using lottery method. The list of eligible women was obtained from registration books of the respective kebeles’ health extension workers. The sample sizes calculations were done using Epi info version 7.

### Data collection tool, procedure, and quality control

A structured questionnaire was used to collect data. Data were gathered on mothers’ age, marital status, place of residence, family income, educational status, occupation, educational status of the husband, occupation of the husband, institutional delivery service utilization, distance from health facility, family size, ANC visit, parity, gravidity, knowledge about dinger signs of labour, reasons for choosing a particular place of birth and decision power on place of delivery. The data collection tool used was a standard tool taken from Ethiopian Demographic and Health Survey (EDHS 2016) questionnaire [7].

The questionnaire was initially prepared in English and translated to the local language Amharic and back translated to English by language experts to check consistency. The questionnaire was pretested on 71 mothers (5% of the total sample size) with similar contexts out of the actual study area. Five diploma graduate nurses supervised by two BSc graduate public health officers collected for the data. Two days training was given to data collectors and supervisors about the objectives and data collection process by the principal investigator. The data were checked for accuracy and consistency on a daily basis. Data cleaning and crosschecking were made by the principal investigator.

### Operational definitions

**Institutional delivery:** is a delivery assisted by skilled birth attendant in the health facility and it had yes or no response.

**Home delivery:** When a mother gave birth at her home or others’ home (neighbor, relatives, or family) or when a birth takes place outside of health institution [17].

**Woman’s decision power:** If a woman decided where to give birth by herself or with her husband jointly.

**Knowledgeable:** was considered a woman who scored above the mean of danger sign knowledge assessment questions and otherwise they were considered as having poor knowledge [18].

**Close to health facility:** was considered if a woman reported to travel < 5 km on foot to reach the health care facility [19].

### Data processing and analysis

Each of the completed copies of the questionnaire was checked for completeness and consistency at the spot. Data were entered in to EPI data version 3.1 and then cleaned and coded before it is exported to SPSS version 20 for analysis. Wealth index was constructed using a principal component analysis (PCA) after having data on household assets, which included a durable asset list, recording the land and animals owned and observing housing materials method. Finally, different significant family asset factors were summed up to categorize individuals into wealth tertiles (low, medium, high). Descriptive statistics, such as proportions, mean with standard deviation, and median were accordingly computed. Both bi-variable and multivariable binary logistic regression analysis were performed. In the bi-variable analysis, variables with *p*-value of < 0.2 [20] were taken to the final multivariable model. Adjusted Odds Ratios (AOR) with 95% Confidence Intervals (CI) were calculated to show the presence and strength of associations. A variable with p-value of less than 0.05 in the multivariable logistic regression model was considered as statistically significant.

## Results

### Socio demographic and economics characteristics

A total of 1424 mothers who gave birth in the past 24 months completed the study with a response rate of 99.9%. The mean age (±SD) of urban and rural mothers were 26.04 (±4.2) and 27.21 (± 4.6) years, respectively. More than half, 414 (58.1%), of the urban and 492 (69.2%) of the rural mothers were within 25 to 39 years of age category. Four hundred three (56.5%) of the urban and the majority, 635(89.3%), of the rural respondents belonged to Oromo ethnic group (Table 1).

**Table 1:** Socio-demographic and economic characteristics of urban and rural mothers who gave birth within the past 2 years in Mana district, Ethiopia, 2017

Variables	Frequency (%)		Total
	Urban	Rural	
Age of the mother			
**15–24**	293(41.1)	210 (29.5)	503 (35.3)
**25–39**	414 (58.1)	492 (69.2)	906 (63.6)
**40–49**	6 (0.8)	9 (1.3)	15 (1.05)
Ethnicity			
**Oromo**	403 (56.5)	635 (89.3)	1038 (73.0)
**Amhara**	141 (19.8)	40 (5.6)	181 (12.7)
**Gurage**	111 (15.6)	13 (1.8)	124 (8.7)
**Tigre**	29 (4.1)	0	29 (2.03)
**Others**	29 (4.1)	23 (3.2)	52 (3.6)
Religion			
**Muslim**	384 (53.9)	645 (90.7)	1029 (72.2)
**Orthodox**	218 (30.6)	58 (8.2)	276 (19.3)
**Protestant**	103 (14.4)	7 (1.0)	110 (7.7)
**Other**	8 (1.1)	1 (1)	9 (0.6)
Educational status of the mother			
**Unable to read and write**	31 (4.3)	366 (51.5)	397 (28.0)
**Can read and write only**	106 (14.9)	163 (22.9)	269 (19.0)
**Primary and above**	576 (80.8)	182 (25.6)	758 (53.2)
Educational status of the husband			
**Unable to read and write**	8 (1.1)	174 (24.5)	182 (13.0)
**Can read and write only**	21 (2.9)	215 (30.2)	236 (16.5)
**Primary and above**	653 (91.6)	317 (44.6)	970 (68.1)
Marital status			
**Married**	662 (92.8)	678 (95.4)	1340 (94.1)
**Single, divorced & widowed**	51 (7.2)	33 (4.6)	84 (6.0)
Occupation			
**Farmer**	26 (3.6)	546 (76.8)	572 (40.1)
**Merchant**	213 (29.9)	71 (10.0)	284 (20.0)
**Government employee**	221 (31.0)	15 (2.1)	236 (16.5)
**Daily laborer**	69 (9.7)	57 (8.0)	126 (8.8)
**Housewife**	174 (24.4)	22 (3.1)	196 (13.7)
**Other**	10 (1.4)	0 (0.0)	10 (0.7)
Wealth quintile			
**Poorest**	125 (17.5)	164 (23.1)	289 (20.2)
**Poorer**	125 (17.5)	117 (16.5)	242 (17.0)
**Middle**	147 (20.6)	151 (21.4)	298 (20.9)
**Richer**	172 (24.1)	133 (18.7)	305 (21.4)
**Richest**	134 (18.8)	141 (19.8)	275 (19.3)

### Maternal and institutional characteristics

The majority, 593 (83.2%), of the urban and 565 (79.5%) of the rural mothers had less than five children. Almost all of the urban, 698 (98.0%), and rural, 699 (98.3%), mothers had at least one ANC follow up, and 195 (27%) of the urban and 462 (65%) of the rural mothers made four and more ANC visits during their last pregnancy. Out of those who attended ANC, 45 (6.3%) of the urban and 76 (10.7%) of the rural mothers reported that they were not informed about place of delivery during their ANC visits. About one-third, 404 (62.0%), of the urban and two-third, 442 (76.2%) of the rural mothers had reported to having decision making power regarding the identification of place of delivery. Regarding their knowledge of the danger signs of labor, 222 (31.1%) of the urban and 82 (11.5%) of the rural mothers had good knowledge, while 697 (97.8%) of the urban and 703 (98.9%) of the rural mothers knew about institutional delivery. Health professionals were the main source of information for 376 (53.6%) urban and 468 (66.6%) rural participants (Table 2).

**Table 2:** Maternal, health facility, and Health facility delivery factors of urban and rural women who gave birth within the past two years in Mana district, Ethiopia, 2017

Variables	Frequency (percent)		Total
	Urban	Rural	
Total number of live births			
**< 5 children**	593 (83.2)	565 (79.5)	1158 (81.3)
**> =5 children**	120 (16.8)	146 (20.5)	266 (18.7)
ANC visit during last pregnancy			
**No**	15 (2.1)	12 (1.7)	27 (2.0)
**Yes**	698 (98.0)	699 (98.3)	1397 (98.1)
Number of ANC visit during last pregnancy			
**< =2 times**	78 (10.9)	30 (4.2)	108 (7.6)
**Three times**	425 (59.6)	207 (29.1)	632 (44.3)
**4 and above**	195 (27.3)	462 (65.0)	657 (46.1)
Informed about place of delivery during last pregnancy			
**No**	45 (6.3)	76 (10.7)	121 (8.5)
**Yes**	659 (92.4)	626 (88.0)	1285 (90.2)
Obstetric complication during last pregnancy			
**No**	679 (95.2)	642 (90.3)	1321 (92.7)
**Yes**	34 (4.8)	68 (9.6)	102 (7.1)
Decision making power			
**Myself**	404 (62.0)	442 (76.2)	846 (68.7)
**Husband**	220 (33.8)	131 (22.6)	351 (28.5)
**Other family members or relatives**	27 (4.1)	7 (1.2)	34 (2.7)
Know about institutional delivery			
**No**	16 (2.2)	8 (1.1)	24 (1.7)
**Yes**	697 (97.8)	703 (98.9)	1400 (98.3)
Any health facility near			
**No**	13 (1.8)	4 (0.6)	17 (1.2)
**Yes**	700 (98.1)	707 (99.4)	1407 (99.0)
Travel time to nearby health facility			
**Less than 30 min**	635 (89.5)	286 (40.2)	921 (64.5)
**More than 30 min**	78 (11.0)	425 (59.7)	503 (35.3)
Mode of travel			
**By foot**	150 (21.3)	317 (45.0)	467 (33.0)
**Motorized**	562 (79.0)	387 (55.0)	949 (67.0)
Delivered at health facility			
**Yes**	651 (91.3)	580 (81.6)	1231 (86.4)
**No**	62 (8.7)	131 (18.4)	193 (13.6)
Reason for not utilizing institutional delivery services			
**High cost in health facility**	4 (6.4)	15 (11.4)	64 (27.0)
**Facility not opened**	5 (8.1)	0	5 (2.1)
**Facility too far/ no transportation**	4 (6.4)	13 (9.9)	17 (7.1)
**Poor quality of service**	2 (3.2)	2 (1.5)	4 (1.7)
**Husband or family did not allow**	17 (27.4)	15 (11.4)	32 (13.4)
**Not necessary**	27 (43.5)	61 (46.5)	88 (37.0)
**Not customary**	3 (4.8)	25 (19.1)	28 (11.7)

### Institutional delivery service utilization

The overall prevalence of institutional delivery was 86.4%. Out of the total respondents, 651(91.3%), of the urban and 580 (81.6%) of the rural mothers gave their last birth at health institutions, and. Among urban mothers who gave birth at home, the main reasons for preferring home delivery were; institutional delivery was not necessary, 23 (37.7%) and husbands or family members did not allow them to give birth at health institutions, 17 (27.9%). Similarly, institutional delivery was not necessary, 61 (46.2%), it was not customary, 25 (18.9%)) and high service cost in the health facilities, 15 (11.4%) were the major reasons provided by rural mothers for preferring home delivery (Table 3).

**Table 3:** Institutional delivery service among urban and rural mothers, who gave birth within the past two years in Mana district, Ethiopia, 2017

Variables	Frequency (%)		Total
	Urban	Rural	
Delivered at health facility			
**Yes**	651 (91.3)	580 (81.6)	1231 (86.4)
**No**	62 (8.7)	131 (18.4)	193 (13.6)
Reason for not utilizing institutional delivery services			
**High cost in health facility**	4 (6.4)	15 (11.4)	64 (27.0)
**Facility not opened**	5 (8.1)	0	5 (2.1)
**Facility too far/ no transportation**	4 (6.4)	13 (9.9)	17 (7.1)
**Poor quality of service**	2 (3.2)	2 (1.5)	4 (1.7)
**Husband or family did not allow**	17 (27.4)	15 (11.4)	32 (13.4)
**Not necessary**	27 (43.5)	61 (46.5)	88 (37.0)
**Not customary**	3 (4.8)	25 (19.1)	28 (11.7)

### Factors associated with institutional delivery

The aim of this study was to compare the prevalence and associated factors of institutional delivery among urban and rural mothers who gave birth in the last 24 months. Unfortunately, the model looked unstable and institutional delivery service utilization among urban and rural mothers was high in both groups as most urban and rural mothers gave birth at health institutions. In addition, information was collected from similar number of urban and rural residents and there was no significant difference between the two groups. Hence, it was realized that abandoning the urban-rural comparison and identifying the factors influencing institutional delivery by combining the two groups was found to be the most appropriate approach.

In the overall combined final multivariate logistic regression analysis, age of the mother, educational status of the husband, wealth index, knowledge of danger signs of labor, travel time to nearby health institutions, and frequency of ANC visits were significantly associated with institutional delivery service utilization. Thus, mothers in the age group of 15–24 were nearly 16 times more likely to deliver at health institutions compared to those in the 35 and above age group, [AOR = 15.71 (95%CI:4.05, 60.9)]. Likewise, mothers whose husbands had primary or above education level were nearly four times more likely to deliver at health institutions than those mothers whose husbands had no formal education, [AOR = 3.84 (95%CI:2.12, 6.98)]. Mothers who had three and more ANC visits were 7 times more likely to give birth at health institutions compared to those who had less than three ANC visits during their last pregnancy, [AOR = 7.36 (95%CI:3.98, 13.61)]. Mothers who were in the richest wealth quantile category were three times more likely to deliver at health institutions than those who were in the poorest wealth quintile category, [AOR = 3.11 (95%CI:1.66, 5.81)].

Mothers whose travel time to nearby health institutions was less than 30 min were 1.8 times more likely to utilize institutional delivery services compared to their counterparts, [AOR = 1.81(95%CI:1.19, 2.74)]. Compared to mothers who had poor knowledge of the danger signs of labor, those mothers who had good knowledge were more likely to utilize institutional delivery services, [AOR = 1.99 (95%CI:1.05, 3.79)] (Table 4).

**Table 4:** Factors associated with institutional delivery service utilization among urban and rural mothers in Mana district, Ethiopia, 2017

Variables	Place of delivery		COR (95%)	AOR (95%)
	Home	Health facility		
Age of mothers				
**15–24**	35 (7.0)	468 (93.0)	15.28 (5.24, 44.64)	15.7 (4.05, 60.9)**
**25–39**	150 (16.5)	756 (83.4)	5.76 (2.06, 16.12)	8.95 (2.51, 31.9)
**40–49**	8 (53.3)	7 (46.7)	1	1
Educational status of the mother				
**Unable to read and write**	88 (22.1)	309 (77.8)	1	1
**Can read & write only**	68 (25.3)	201 (74.7)	0.84 (0.58, 1.21)	0.69 (0.42, 1.15)
**Primary & above**	37 (4.9)	721 (95.1)	5.55 (3.69, 8.33)	1.81 (0.96, 3.41)
Educational status of the husband				
**Unable to read & write**	53 (29.1)	129 (70.9)	1	1
**Can read & write only**	48 (20.3)	188 (79.7)	1.61 (1.03, 2.52)	1.62 (0.95, 2.71)
**Primary and above**	81 (8.3)	889 (91.6)	4.51 (3.05, 6.67)	3.84 (2.12, 6.98)**
Number of ANC follow up				
**< =2 times**	45 (41.7)	63 (58.3)	1	1
**Three times**	52 (8.2)	580 (91.8)	7.97 (4.94, 12.83)	7.36 (3.98, 13.61)**
**4 and above**	73 (11.1)	584 (88.8)	5.71 (3.63, 8.99)	9.55 (5.05, 18.07)
Knowledge on the danger sign of labor				
**Poor**	177 (15.8)	943 (84.2)	1	1
**Good**	16 (5.2)	288 (94.7)	3.38 (1.99, 5.73)	1.99 (1.05, 3.79)*
Wealth index				
**Poorest**	75 (26.0)	214 (74.0)	1	1
**Poorer**	27 (11.2)	215 (88.8)	2.79 (1.73, 4.51)	2.42 (1.304, 4.48)*
**Middle**	35 (11.7)	263 (88.3)	2.63 (1.69, 4.09)	1.81 (1.03, 3.18)*
**Richer**	29 (9.5)	276 (90.5)	3.34 (2.09, 5.31)	2.81 (1.53, 5.14)*
**Richest**	24 (8.7)	251 (91.2)	3.67 (2.24, 6.01)	3.105 (1.66, 5.81)**
Mode of travel				
**By foot**	80 (17.1)	387 (82.9)	1	1
**Motorized**	105 (11.0)	844 (89.0)	1.66 (1.21, 2.27)	1.43 (0.94, 2.19)
Travel time to nearby health facility				
**Less than 30 min**	82 (8.9)	839 (91.1)	2.87 (2.11, 3.91)	1.81 (1.19, 2.74)
**More than 30 min**	111 (22.1)	392 (78.0)	1	1

NP: * = *p* value < 0.05, ** = *p* value< 0.001

## Discussion

The aim of this study was to assess the magnitude of institutional delivery and its associated factors among urban and rural mothers who gave birth in Mana district, Ethiopia, in the past 24 months.

The result of the current study showed that 86.4% of mothers gave their recent birth at health institutions. This finding is approximately in line with a study done in Ethiopia, which revealed that 81% of mothers gave birth at health institutions [21].

On the other hand, our result is higher than the previous local studies’ findings in Ethiopia, such as Dodota (62.4%), Assosa (72.5%), Bench Maji (78.3%), and Mirab Abaya districts (54%) [22–25]. It is also higher conducted in Zambia (42.8%) and Tanzania (54%) concerning the utilization of institutional delivery services [25, 26].

The observed discrepancy between studies might be due to difference in study population, sample sizes, study period and sociocultural characteristics. In addition, the reason could probably be due to more attention has been given to increase the uptake of institutional delivery services more than ever in Ethiopia. Recently, ministry of health of Ethiopia is providing ANC and institutional delivery services for free. Health facilities have their own ambulance services. Hence, these freely available services might increase the proportion of mothers who gave birth at the health institutions [27].

Almost all of the urban, 698 (98.0%), and rural, 699 (98.3%), mothers had at least one ANC follow up, and 195 (27%) of the urban and 462 (65%) of the rural mothers made four and more ANC visits during their last pregnancy. This study shows more proportion of mothers who made four and above ANC visits were from rural once. This might be related to more attention and efforts has been given to rural communities by the government and other stack holders. In addition, in Ethiopia, women to women support group and women development army with the aim and channel of community participation are engaged in maternal and child health issues together with health extension workers [28] These groups are community based initiative where rural women form groups, meet regularly, discussing on maternal health issues and come up with locally available solutions. They held conferences per month, shared their experiences and advice younger pregnant women, this in turn may help them to have more ANC visits and decision making power than the rural mothers [29].

In this study, younger mothers (below 40 years) were more likely to use institutional delivery services than older mothers (above 40 years). This finding is parallel with other studies conducted in Ethiopia [26, 30], and abroad in Tanzania and Nepal which revealed that older women tended to deliver at home compared to younger once [31, 32]. This might be due to the fact that younger mothers had no previous experiences in giving births and might have great fear of complications relating to pregnancy, labor and child birth. On the other hand, older mothers had repeated birth experiences and thus, they might not need to be assisted by skilled health professionals.

The number of ANC visits tended to increase the utilization of institutional delivery services. This finding is in line with those of other studies conducted in different parts of Ethiopia [33–35]. This can be partially explained by the fact that ANC visit is one of the contact points with health professionals and the more the ANC visits done, the better the probability of getting adequate information about danger signs and complications related to pregnancy and the importance of delivering at the health institutions. This implies that making repeated ANC visits has a paramount importance to increase the utilization of institutional delivery services.

Husband’s educational status was the other factor significantly associated with institutional delivery service utilization. Mothers whose husbands had at least primary school education were nearly 4 times more likely to utilize institutional delivery services. This finding is in agreement with studies done in a different places in Ethiopia [21, 36]. The possible explanation for this evidence could be, for example, literate husbands; a) are more likely to discuss with their wives on issues, including pregnancy and labour and reach at a consensus, b) may give to their wives’ freedom to decide the place of delivery, and c) may even be the one who insist and support the mother to have a health facility delivery.

A significant association was also observed between wealth index and institutional delivery service utilization. Mothers who were in the rich and richest wealth quintiles were more likely to deliver at heath institutions compared to those who were in the poorest wealth quintile. This result is in agreement with other local studies in Ethiopia that showed institutional delivery service utilization was higher among women in the richest quintile than those in the lower economic ranks [26, 37].. This may be due to the fact that mothers who belong to poor families may not be able to afford transportation and other indirect costs. Besides, mothers in the low socioeconomic status may have lower access to health information. This finding suggests that improved economic status has a vital role to enhance utilization of institutional delivery service.

Mothers who had good knowledge about the danger signs of labor were about two times more likely to utilize health institution delivery services than their counterparts. This finding was consistent with those of other studies done in Ethiopia and Tanzania which showed the knowledge of mothers about pregnancy and labor risk factors was two and three times more likely to encourage mothers to deliver at health institutions [14, 38]. This could probably be due to the fact that being aware of the danger signs of labor enables mothers to predict the upcoming devastating consequences, which may in turn be a pushing factor for mothers to visit health institutions to give birth. This implies that providing adequate information during ANC and other contact points have a great positive impact to improve health institution delivery. Therefore, creating awareness on danger signs of pregnancy, labor, child birth and place of delivery is necessary to encourage mothers to prefer delivering at the health institutions.

The other important factor that was identified by this study was the impact of traveling time (distance) needed to reach nearby health institutions. Women who had to walk more than 5 km to reach to the health institution were less likely to give birth at health institution than those who had to travel less than 5 km or less. This finding is in line with those of other similar studies done in Ethiopia and other developing countries [39–41]. It might be related to easily accessible health institutions may increase the chances of mothers to use it during prenatal periods and at a time of delivery. This finding suggests that making health institutions closer to and easily accessible by the communities is very crucial in order to enable more mothers to give birth at the health institutions.

### Limitations of the study

This study is not without limitations. Firstly, even though, we provided an intensive training for data collectors and supervisors, and a pretested and structured questionnaire was used, help mothers to recall past events the finding might have been affected by recall bias as some of the information collected was based on events which has happened sometime in the past. As a result, it is likely that our estimates may overestimated the magnitude of institutional delivery. Secondly, information regarding health professionals’ hospitality, and health institutions’ infrastructure information regarding health professionals’ hospitality, quality of health institutions and behavioral factors of the participants were not collected.

## Conclusion

Utilization of institutional delivery services was high among mothers who gave birth within the last 24 months. Increased number of antenatal care visits, partners’ better educational status, mothers’ good knowledge on dangers signs of labour, increased wealth index, and lesser travelling distance to reach to the health facility have significantly increased the utilization of institutional delivery services. Therefore, attending ANC and maintain frequent visits as per the minimum recommendation by the WHO will a positive impact on pregnant mothers to give birth at health institutions. Working on expansion of health institutions and addressing infrastructure issues will significantly contribute to increase the number of mothers giving birth at health institutions. Promoting husbands’ education will also be helpful to encourage mothers to deliver at health institutions. Furthermore, it is also very crucial to empower women and awareness creation.

## Data Availability

Data will be available upon request from the corresponding author.

## Abbreviations

ANC: Antenatal care
EDHS: Ethiopian demographic and health survey
SPSS: Statistical package for social science
